# Influence of Dietary Garlic (*Allium sativum*) and/or Ascorbic Acid on Performance, Feed Utilization, Body Composition and Hemato-Biochemical Parameters of Juvenile Asian Sea Bass (*Lates calcarifer*)

**DOI:** 10.3390/ani10122396

**Published:** 2020-12-15

**Authors:** Abdelwahab M. Abdelwahab, Sabry M. El-Bahr, Sami Al-Khamees

**Affiliations:** 1 Department of Animal and Fish Production, College of Agricultural and Food Sciences, King Faisal University, Al-Ahsa, Al-Hufof 31982, Saudi Arabia; amsaid@kfu.edu.sa (A.M.A.); salkhamees@kfu.edu.sa (S.A.-K.); 2 Department of Animal Production, Faculty of Agriculture, Fayoum University, Fayum 63514, Egypt; 3 Department of Biomedical Sciences, College of Veterinary Medicine, King Faisal University, Al-Ahsa, Al-Hufof 31982, Saudi Arabia; 4 Department of Biochemistry, Faculty of Veterinary Medicine, Alexandria University, Alexandria 21526, Egypt

**Keywords:** garlic, ascorbic acid, juvenile barramundi, growth, blood

## Abstract

**Simple Summary:**

Ascorbic acid and garlic have been used in several studies as enhancers or promoters of growth performance and health conditions in mammalian species. However, few studies have been performed in fish. In this regard, this study aimed to evaluate the effects of garlic and/or ascorbic acid on growth performance, feed utilization, chemical body composition, and hemato-biochemical parameters of juvenile Asian sea bass. The results demonstrated that dietary supplementation of garlic alone (40 g/kg diet) was highly effective in improving the studied parameters in comparison with that of ascorbic acid alone or a mixture of garlic (20 g/kg diet) and ascorbic acid (0.75 g/kg diet).

**Abstract:**

The current study investigated effects of garlic (*Allium sativum*) and/or ascorbic acid on growth performance, feed utilization, biochemical body composition, and hemato-biochemical parameters of juvenile Asian sea bass. A total of 600 fish (43.14 ± 0.23 g body weight) were divided into four groups. Fish in the first group were fed basal diet and served as a control group. Fish in groups 2, 3 and 4 were fed a basal diet mixed with garlic (40 g/kg diet), ascorbic acid (1.5 g/kg diet), or garlic (20 g/kg diet)/ascorbic acid (0.75 g/kg diet) mixture, respectively, for 12 weeks. A significant (*p* < 0.05) increase was observed in growth performance, feed utilization, and chemical body composition in fish fed garlic alone in comparison with the control and other treated groups. All hematological indices, biochemical parameters, and survival rate were not changed significantly (*p* > 0.05) in all groups throughout the experimental period when compared with the control. Total cholesterol and feed conversion ratio were significantly (*p* < 0.05) decreased in fish fed garlic alone in comparison to the control and other treated groups. Conclusively, dietary supplementation of garlic alone (40 g/kg diet) was highly effective in improving most of the studied parameters in comparison with that of ascorbic acid alone or a mixture of garlic (20 g/kg diet) and ascorbic acid (0.75 g/kg diet).

## 1. Introduction

The increase of aquaculture fish production is required to meet the growing demand of humans for animal protein due to the significant increase of human population not only in Saudi Arabia but also worldwide [1]. Asian seabass (*Lates calcarifer*) is considered an economically desirable fish food in Asia and Pacific regions because of its flesh texture and taste. It is commercially cultivated in Asia, in both brackish water and freshwater ponds. Asian seabass culture enterprise in Southeast Asia is one of the most dynamic and potentially profitable segments of the fish farming industry. It has become an attractive commodity to both small- and large-scale aquaculture enterprises [2,3]. Stressors are common problems found upon intensive fish culture practices such as poor water quality, overcrowding, transport, handling, and size grading. In addition, fish diseases in intensive farming negatively effect aquaculture productivity and sustainability, with a subsequent impact on food security and human health [4,5]. Several practices have been proposed to improve growth performance and health conditions of fish cultivated in intensive farms such as improved husbandry, nutrition and water quality, antibiotic usage [6,7], and interfering with bacterial invasion strategies [8]. Enhancement of immune system of aquaculture fish is perhaps the most interesting approach towards preventing fish diseases. This can be achieved through antibiotic application, which is considered the most effective agent. On the other hand, the risk of generating resistant pathogens and environmental pollution has arisen due to usage of antibiotics and chemotherapeutics for combating fish diseases. In addition, the commercial antibiotics and chemotherapeutic agents available are highly specific against particular pathogens but are expensive for fish farmers [9]. Immune stimulants, on the other hand, enhance fish non-specific immune response [10]. There are several biological compounds that have been shown to enhance the non-specific immune system of cultivated fish [4,11]. Several medicinal plants and their components or extracts have several activities that improve growth performance, blood parameters, and health conditions, and have been explored in different species, including fish [12,13,14,15,16,17]. Ascorbic acid and garlic were used in several studies as enhancers or promoters of growth performance and health conditions in mammalian species [18,19], although few studies have been performed in fish [20,21,22]. Ascorbic acid is a natural organic compound that plays multifunctional biological roles, including antioxidant [23], immune regulation [24], anti-stress [18], and gene regulation [25]. Dietary inadequacy of ascorbic acid for fish has been reported in several studies. Ascorbic acid deficiencies decreased growth and survival rate in fish in addition to abnormalities of cartilage, skeletal tissues, and pigmentation [26]. Ascorbic acid deficiency symptoms include anemia, low growth, and impaired collagen formation [27]. Therefore, supplementation of ascorbic acid to fish is necessitated to improve abnormal characters of cultivated fish. Garlic (*Allium sativum*) is a plant with antibiotic effects [28] that increases macrophage and the other immune cells in the animal body [29]. Garlic acts as an immune stimulant and disease control agent in aquatic organisms and improves feeding indices and body chemical composition [30]. Garlic caused enhancement of lysozyme activity in hybrid tilapia (*Oreochromis niloticus* × *Oreochromis aureus*) [31]. Allicin is a sulfur compound that has an important role as an antibacterial, antifungal, and antioxidant material. Amino acids, minerals, vitamins, and flavonoids are the other compounds in garlic [32]. Therefore, the current study investigated effects of garlic (*Allium sativum*) and/or ascorbic acid on growth performance, feed utilization, chemical body composition, and hemato-biochemical parameters of Juvenile Asian sea bass.

## 2. Materials and Methods

### 2.1. Feed Additives

Ascorbic acid (L-ascorbic acid, AA) was supplied from Sigma Aldrich Company (St. Louis, MO, USA). Natural garlic was prepared upon removal of the dry outer covers, dried, and ground to powder.

### 2.2. Experimental Design

The study was carried out according to procedures approved by the Ethics Committee of Animal Experimentation of King Faisal University, Saudi Arabia. Barramundi (Asian *Lates calcarifer* Bloch) were obtained from a Saudi fish farm. The fish were kept during the experimental period under cycle-controlled conditions of 12 h light and 12 h dark periods. The controlled Celsius temperature (°C) and relative humidity (RH%) during the experimental period were, respectively, 26.0 ± 3.0 °C and 58.0 ± 10.0%. The recorded parameters of water quality during the study were 7.3–8.2 mg/L dissolved oxygen, 6.3–7.1 pH, 26–28 °C temperature, and <0.003 mg/L total ammonia. Fish were allowed to adapt for 2 weeks and were weighed using digital balance (Chyo Petit Balance MK-500C-Japan) (43.14 ± 0.23 g); then, randomly, a total number of 600 fish (43.14 ± 0.23 g body weight) were divided into 4 groups. The fish were distributed at a stocking density of 50 fish per aquarium, and each group was represented by 3 aquaria as replicates. Fish in the first group were fed a basal diet and served as the control group. Fish in the groups 2, 3, and 4 were fed diets supplemented with garlic (40 g/kg diet) [33], ascorbic acid (1.5 g/kg diet) [34], or a mixture of garlic (20 g/kg diet) and ascorbic acid (0.75 g/kg diet), respectively. The diets were formulated and prepared to meet the nutrient requirements of juvenile barramundi fish [35]. The quantities of diets were given on the basis of live body weight and were readjusted according to changes in body weight gain. The diets were given twice a day for 12 weeks. Ingredients (g/kg) and chemical composition percentage of control and treated formulated diets used during the experimental period are presented in Table 1. Throughout the experimental period, fish were healthy (regular movement, absence of any lesions, and ability to feed).

### 2.3. Growth Performance and Feed Utilization

Body weight of all fish were recorded at the start and the end of the experiment period (84 days) in order to calculate weight gain (WG), daily weight gain (DWG), and specific growth rate (SGR) using digital balance (Chyo Petit Balance MK-500c-Japan) (Table 2). Fish were anesthetized using 0.1 g/L tricainemethane sulfonate and dried before weighing. Body weight gain was determined by subtracting final body weight from initial body weight. Tanks were cleaned before feeding using symphony system. Diets were offered twice a day at 7:00 a.m. and 2:30 p.m. Feed efficiency was calculated by dividing feed intake by body weight gain (Table 3). The gross energy content was calculated using the factors of 5.64 Kcal/g protein, 9.45 kcal/g lipids, and 4.10 kcal/g diet [30]. The parameters of growth performance and feed utilization were calculated using the following equations:

- Specific growth rate (SGR) = InWt-ln WO/T × 100, where In = natural logarithm; WO = initial original weight; Wt = final weight; and T = time in days. Feed conversion ratio (FCR) = Total dry feed consumed (g)/total wet weight gained (g). Feed efficiency ratio (FER) = Live weight gained (g)/Dry feed given (g) × 100. Protein efficiency ratio (PER) = Wet weight gained (g)/Amount of protein fed (g).
- PPV, protein productive value, % = {(retained protein (g)/protein intake (g)} ×100.
- EER, energy efficiency ratio, = weight gain (g)/energy intake (kcal).
- EPV, energy productive value, % = {(retained energy (kcal)/energy intake (kcal)} × 100.

### 2.4. Hematological and Biochemical Analysis

Blood samples were collected via the caudal vein of fish in each group. Blood samples were decanted in tubes containing 0.14% anticoagulant (EDTA K3, Australia) and transported via ice box to biochemistry laboratory within 30 min for estimation of total erythrocytic count (TEC), total leucocytic count (TLC), and packed cell volume (PCV) [36]. Hemoglobin (Hb) was assessed [37], and differential leucocytic count was determined [38,39]. The mean corpuscular volume (MCV), mean corpuscular hemoglobin (MCH), and mean corpuscular hemoglobin concentration (MCHC) were calculated and expressed as fL, pictogram (pg/cell), and g L^−1^, respectively [40]. Similarly, blood samples were collected without anticoagulant for serum separation [41]. The obtained sera were used for spectrophotometric determination of the activities of aspartate transaminase (AST) and alanine transaminase (ALT) [42]. In addition, serum glucose, total protein, albumin, and globulin values were determined spectrophotometrically, as per [43,44,45,46], respectively. Serum blood urea nitrogen (BUN), and uric acid and bilirubin were determined as per [47,48], respectively. Furthermore, the obtained sera were total cholesterol by use of enzymatic method of spin react kits [49]. Calcium, phosphorus, and magnesium were determined by using commercial kits on chemistry analyzer according to the manufacturer instructions.

### 2.5. Chemical Analysis of Feed Ingredients and Fish Body Composition

Ingredients of diets and fish body samples were dried at 70 °C in an air oven for constant weight. Both diets and fish samples were ground and analyzed for determination of dry matter (DM), organic matter (OM), crude protein (CP), crude fibers (CF), and ether extract (EE) components [50,51] (Table 1 and Table 4).

### 2.6. Statistical Analysis

Statistical analysis was performed according to general linear model (GLM) of SAS program (2008) [52]. Differences between control and garlic (40 g/kg diet), ascorbic acid (1.5 g/kg diet), and both garlic and ascorbic acid (20 g/kg and 0.75 g/kg diet, respectively) groups were tested in growth performances, feed utilization, chemical body composition of fish, and metabolic and blood profiles by one-way ANOVA. Duncan’s multiple range test [53] was used to explore the effect of groups. The data were presented as mean ± standard error of means (SEM) and the level of significance was set at *p* < 0.05. Statistical model was Yi j = µ + Ti + Eij, where Yij = the experimental observation ij, µ = the overall mean, Ti = the effect due to groups i, and Eij = the experimental error.

## 3. Results

### 3.1. Growth Performance, Survival Rate, and Feed Utilization

Data summarized in Table 2 showed a significant (*p* < 0.05) increase in final live wet body mass (g/fish), body weight gain (BWG; g/fish), body weight gain percentage (BWG%), daily gain (g/fish), and specific growth rate percentage (SGR%) in fish fed garlic alone compared to control and other treated groups, which remained comparable to each other (*p* > 0.05). Moreover, no significant differences (*p* < 0.05) were obtained between all groups with regards to the survival rate. The current findings (Table 3) indicated that feed intake (FI), feed efficiency (FE), protein efficiency ratio (PER), and energy efficiency ratio (EER) were significantly (*p* < 0.05) increased, whereas feed conversion ratio (FCR) was decreased significantly in fish fed garlic alone in comparison with control and other treated groups, which remained comparable to each other (*p* > 0.05).

### 3.2. Chemical Body Composition

Dry matter (DM), crude protein (CP), and ash parameters were significantly (*p* < 0.05) increased in fish fed garlic either alone or in a combination with ascorbic acid in comparison with control and ascorbic acid-treated fish (Table 4). Ether extract (EE) and gross energy (GE) remained unchanged significantly in all experimental groups in comparison with the control (Table 4).

### 3.3. Hematological and Biochemical Parameters

The obtained results demonstrated that TEC, Hb, PCV, MCV, MCH, and MCHC were not changed significantly (*p* > 0.05) in all groups throughout the experimental period when compared with the control (Table 5). All biochemical parameters related to glucose and protein metabolism (total protein, albumin, globulin, and albumin/globulin ratio) remained unchanged significantly (*p* > 0.05) in all experimental groups in comparison with the control (Table 6). Garlic and/or ascorbic acid did not disturb liver and kidney functions, as reflected by unchanged activities of estimated liver enzymes (ALT and AST) and kidney function values (BUN, uric acid, and creatinine; Table 6). Total cholesterol was significantly (*p* < 0.05) decreased in fish fed garlic alone in comparison with the control and other treated groups (Table 6). Moreover, the electrolyte balance was not altered in all treated groups, as reflected by unchanged (*p* > 0.05) values of calcium, phosphorus, and magnesium (Table 6).

## 4. Discussion

The doses of garlic and ascorbic acid were given as recommended in previous studies [33,34]. In the present study, the significant increase in growth (BWG and SGR), feed utilization (FI, FE, PER, and EER), and chemical body composition (CP and EE) observed in fish fed crude garlic (40.0/kg diet) diets agree with an earlier study [21] and disagree with other works that used different dietary garlic extracts [54,55,56]. A significant improvement of growth and feed utilization parameters was reported in Nile tilapia fish fed a diet supplemented with garlic crude powder [57]. Significant improvement in parameters of growth performance and feed utilization parameters were similarly reported in Asian sea bass (*Lates calcarifer*) [58], Sterlet sturgeon (*Acipenser rutheni*) [59], orange-spotted grouper (*Epinephelus coioides*) [60], and Caspian roach (*Rutilus rutilus*) [61]. The responses of different levels of garlic supplementation on growth and feed utilization were dose-dependent, and poor growth was observed at high levels in most cases [58,60,61], as demonstrated in the current study.

Although dietary herbs could change some hematological and biochemical indices of the fish, as reported earlier [62], this was not the case in the current study because TEC, Hb, PCV, MCV, MCH, and MCHC remained significantly unchanged in all groups in comparison to the control. In the past year, [63] highlighted the use of hematological parameters as an important tool for aquaculture in the nutritional status and diagnosis of disease of fish. Different types of feed can influence the hematological parameters in teleost. Similarly, serum total protein, glucose, albumin, globulin, ALT, AST, BUN, uric acid, creatinine, calcium, phosphorus, and magnesium were not changed significantly in fish fed garlic and/or ascorbic acid in comparison to the control. The estimated hematological parameters in the current study were comparable to that measured earlier in the same fish species [40]. The unchanged hematological values in fish fed garlic and/or ascorbic acid in comparison to the control indicated the safety of these feed additives for hemopiosis. Similar results were obtained on hematological parameters in juvenile golden shiner [64], sturgeon hybrid [65], and Atlantic halibut [66] fed with different levels of ascorbic acid. A contrary earlier work indicated that garlic increased the TEC, HB, and PCV before challenge with bacterial infection and subsided post-challenge [58]. Therefore, the unchanged hematological parameters of Asian sea bass fish fed either garlic and/or ascorbic acid may indicate good health state of fish reared in the system of the current study. The estimated biochemical parameters in the current study were comparable to that measured earlier in the same fish species [40]. The unchanged level of albumin, globulin, and A/G ratio, along with unchanged ALT and AST indicated that the studied doses of garlic and/or ascorbic acid were safe for Asian sea bass fish, as liver function was not disturbed. Moreover, the unchanged level of BUN, uric acid, and creatinine in Asian sea bass fish fed garlic and/or ascorbic acid in comparison to the control indicated that kidney function of these fish was not disturbed. On the contrary, previous work [58] indicted a significant increase in total protein, albumin, globulin, and albumin/globulin ratio in Asian sea bass fish fed different doses of garlic. The confliction between the current and previous study [58] concerning protein metabolism may be attributed to the different ages and weights of the fish used. In the current study, juvenile Asian sea bass fish were used, however, in the previous work [58], Asian sea bass fingerlings were used instead. High ascorbic acid concentrations could stimulate protein production (total protein, albumin, and globulin) in fish, suggesting an important role of the vitamins in the modulation of plasma proteins [67]. However, our study showed that these parameters were not significantly affected by dietary ascorbic acid levels in Asian sea bass fish. Similar results were obtained earlier for hybrid striped bass (*Piaractus mesopotamicus*) [68] and for golden shiner [64], all fed diets supplemented with different levels of ascorbic acid. The hypocholestrolemic effect of garlic agrees with previous work in the same fish species [58,69,70]. The reduction in cholesterol may have enhanced the cardiovascular activity of fish, thus reflecting beneficial effect in term of wellbeing of fish [58].

Growth performance reflected in the significant increase (*p* < 0.05) of CP and significant decrease of EE in fish fed garlic, which may be attributed to its ability to promote lipid metabolism, leaves spare protein for growth and leads to the repression of lipid accumulation [71]. As a result, quality of fish muscle would be improved (low lipid and high crude protein) [59], as demonstrated in the present study. Garlic induced a significant reduction in total cholesterol, low-density lipoprotein, and blood pressure [72]. The consumption of garlic powder in hamsters decreased the accumulated lipid in liver and increased the excretion of total bile acids [73]. The improvement of growth, feed utilization, and body health due to garlic supplementation could be attributed to many factors. Garlic has been known to be one of the earliest medicinal plants, containing several sulfur-containing compounds (aliin, allicin, ajoene, and others), enzymes (allinase, peroxidases, myrosinase, and others), volatile oils, amino acids (arginine and others), and trace minerals (selenium and others) [27]. Such aforementioned components of garlic make it advantageous in terms of improvement in growth performance and body condition health [74,75]. Our study reported similar growth, feed utilization, and body composition upon ascorbic acid supplementation in comparison to the control, which might be attributable to the level of ascorbic acid or fish species. Other studies indicated improved feed conversion, growth performance, survival rate, immune functions [76,77], and health [75] upon ascorbic acid supplementation. The combination of garlic (20 g/kg diet) and ascorbic acid (0.75 g/kg) improved (*p* > 0.05) body composition in comparison to the control or to ascorbic acid supplementation alone. Such improvement may be attributable to the synergism effect of both garlic and ascorbic acid.

## 5. Conclusions

In conclusion, dietary supplementation of garlic alone (40 g/kg diet) was highly effective in improving the studied parameters in comparison with that of ascorbic acid alone or a mixture of garlic (20 g/kg diet) and ascorbic acid (0.75 g/kg diet). Further studies are needed to investigate the possible role of the hormesis of the studied doses of garlic.

## Figures and Tables

**Table 1:** Formulation and chemical composition of the experimental groups (g/kg feed in dry matter) of Asian seabass (*Lates calcarifer*).

Ingredients (g/kg Feed)	Groups			
	Group 1	Group 2	Group 3	Group 4
Fish meal (65%)	481.40	481.40	481.40	481.40
Soybean meal (48%)	296.60	290.50	296.80	293.70
Yellow corn	127.00	93.10	125.30	109.15
Fish oil	70.00	70.00	70.00	70.00
Salt (sodium chloride)	5.000	5.000	5.000	5.000
Dicalcium phosphate	5.000	5.000	5.000	5.000
Vitamin and mineral premix	10.00	10.00	10.00	10.00
Limestone	5.000	5.000	5.000	5.000
Garlic	0.00	40.0	0.00	20.00
Ascorbic acid	0.00	0.00	1.50	00.75
Total	1000	1000	1000	1000
**Proximate composition**				
Dry matter (DM, g/kg wet weight)	915.28	917.45	915.45	916.45
Crude protein (g/kg DM)	524.46	523.22	524.34	523.80
Ether extract (g/kg DM)	115.22	113.98	115.14	114.56
Crude fiber (g/kg DM)	23.65	24.33	23.60	23.97
Nitrogen-free extract (g/kg DM)	203.25	204.36	201.90	203.11
Crude ash (g/kg DM)	133.43	134.11	135.03	134.57
Energy (kcal/g DM)	48.60	48.46	48.53	48.49

Group 1: basal diet without any additives; group 2: basal diet mixed with garlic (40 g/kg diet); group 3: basal diet mixed with ascorbic acid (1.5 g/kg diet); group 4: basal diet mixed with a combination of both garlic (20 g/kg diet) and ascorbic acid (0.75 g/kg diet).

**Table 2:** Growth performance and survival rate of Asian seabass *(Lates calcarifer)* fed diets supplemented with garlic and/or ascorbic acid.

Parameters	Unit	Groups			
		Group 1	Group 2	Group 3	Group 4
Initial body weight	g/fish	42.98 ± 0.50	42.70 ± 0.60	43.57 ± 0.56	43.30 ± 0.56
Final body weight	g/fish	100.60 ± 0.47 ^b^	119.24 ± 7.14 ^a^	99.96 ± 2.12 ^b^	107.29 ± 5.25 ^b^
Body weight gain	g/fish	57.63 ± 0.35 ^b^	76.54 ± 7.09 ^a^	56.39 ± 1.56 ^b^	63.99 ± 5.81 ^b^
Body weight gain	%	134.12 ± 2.08 ^b^	179.21 ± 16.35 ^a^	129.39 ± 1.93 ^b^	148.17 ± 15.32 ^b^
Daily weight gain	g/fish	0.69 ± 0.003 ^b^	0.91 ± 0.08 ^a^	0.67 ± 0.02 ^b^	0.76 ± 0.07 ^b^
SGR	%	1.01 ± 0.01 ^b^	1.22 ± 0.07 ^a^	0.99 ± 0.01^b^	1.08 ± 0.0 ^b^
Relative SGR	%	100.00	102.79	98.02	106.93
Survival rate	%	98.89 ± 1.11	95.56 ± 4.44	97.78 ± 2.22	96.67 ± 3.33

Values are mean ± standard error (SE) of three replicates of fish (*n* = 3). ^a,b^ values with different superscripts in the same row significantly differed at *p* < 0.05. Group 1: basal diet without any additives; group 2: basal diet mixed with garlic (40 g/kg diet); group 3: basal diet mixed with ascorbic acid (1.5 g/kg diet); group 4: basal diet mixed with a combination of both garlic (20 g/kg diet) and ascorbic acid (0.75 g/kg diet). SGR: specific growth rate.

**Table 3:** Feed utilization of Asian seabass *(Lates calcarifer)* fed diets supplemented with garlic and/or ascorbic acid.

Items	Unit	Groups			
		Group 1	Group 2	Group 3	Group 4
Feed intake	g/fish	140.43 ± 3.66 ^b^	151.42 ± 4.19 ^a^	142.07 ± 0.91 ^b^	141.52 ± 2.62 ^b^
DFI	g/fish/day	2.83 ± 0.07	2.72 ± 0.05	2.87 ± 0.03	2.73 ± 0.03
FE	%	43.50 ± 1.37 ^b^	53.19 ± 3.48 ^a^	41.92 ± 0.89 ^b^	47.48 ± 3.44 ^b^
FCR	ratio	2.44 ± 0.08 ^a^	2.00 ± 0.03 ^b^	2.52 ± 0.05 ^a^	2.24 ± 0.17 ^a^
PER	ratio	0.79 ± 0.02 ^b^	0.98 ± 0.07 ^a^	0.76 ± 0.02 ^b^	0.88 ± 0.06 ^b^
PPV	value	19.91 ± 0.49	20.51 ± 0.87	18.54 ± 0.64	18.66 ± 1.17
EER	ratio	8.48 ± 0.27 ^b^	10.30 ± 0.67 ^a^	8.04 ± 0.17 ^b^	9.21 ± 0.67 ^b^
EPV	%	18.89 ± 0.07	20.10 ± 1.66	19.58 ± 0.83	19.61 ± 1.24

Values are mean ± standard error (SE) of three replicates of fish (*n* = 3). ^a,b^ values with different superscripts in the same row significantly differed at *p* < 0.05. Group 1: basal diet without any additives; group 2: basal diet mixed with garlic (40 g/kg diet); group 3: basal diet mixed with ascorbic acid (1.5 g/kg diet); group 4: basal diet mixed with a combination of both garlic (20 g/kg diet) and ascorbic acid (0.75 g/kg diet). DFI: daily feed intake; FE: feed efficiency; FCR: feed conversion ratio; PER: protein efficiency ratio; PPV: productive protein value; EER: energy efficiency ratio; EPV: energy productive value.

**Table 4:** Chemical body composition of Asian seabass *(Lates calcarifer)* fed the experimental diets (wet matter basis).

Parameters	Units	Groups			
		Group 1	Group 2	Group 3	Group 4
Moisture	g	710.40 ± 2.54 ^a^	687.77 ± 4.53 ^b^	702.35 ± 2.86 ^a^	679.90 ± 3.64 ^b^
DM	g	289.60 ± 2.54 ^b^	312.23 ± 4.53 ^a^	297.65 ± 2.86 ^b^	320.10 ± 3.64 ^a^
CP	g	182.57 ± 1.59 ^b^	201.67 ± 0.88 ^a^	179.77 ± 1.19 ^b^	195.30 ± 2.28 ^a^
EE	g	63.80 ± 5.20	63.33 ± 3.55	72.67 ± 1.71	78.20 ± 2.36
Ash	g	43.23 ± 1.18 ^b^	47.23 ± 0.23 ^a^	44.03 ± 1.07 ^b^	46.60 ± 0.86 ^a^
GE	kcal/g	16.32 ± 0.40	17.35 ± 0.38	17.00 ± 0.16	18.40 ± 0.34

Values are mean ± standard error (SE) of three replicates of fish (*n* = 3). ^a,b^ values with different superscripts in the same row significantly differed at *p* < 0.05. Group 1: basal diet without any additives; group 2: basal diet mixed with garlic (40 g/kg diet); group 3: basal diet mixed with ascorbic acid (1.5 g/kg diet); group 4: basal diet mixed with a combination of both garlic (20 g/kg diet) and ascorbic acid (0.75 g/kg diet). DM: dry matter; CP: crude protein; EE: ether extract; GE: gross energy.

**Table 5:** Influence of garlic and/or ascorbic acid on biochemical parameters of juvenile barramundi (Asian sea bass, *Lates calcarifer* Bloch) fish.

Parameters	Groups			
	Group 1	Group 2	Group 3	Group 4
TEC mm × 10^6^	3.2 ± 0.1	3.3 ± 0.1	3.0 ± 0.3	3.1 ± 0.5
PCV (%)	26.0 ± 0.8	27.0 ± 1.0	26.0 ± 1.0	27.1 ± 1.1
Hb (%)	10.1 ± 0.1	10.1 ± 0.2	10.2 ± 0.3	10.1 ± 0.1
MCV (fL)	83.9 ± 1.1	81.8 ± 3.5	86.7 ± 2.5	87.4 ± 4.0
MCH (pg/cell)	31.6 ± 1.0	30.6 ± 1.7	34.0 ± 3.2	32.6 ± 1.2
MCHC (g/L)	3.88 ± 0.5	3.74 ± 0.6	3.90 ± 1.1	3.74 ± 1.0

Values are mean ± standard error (SE) of three replicates of fish (*n* = 3). ^a,b^ values with different superscripts in the same row significantly differed at *p* < 0.05. Group 1: basal diet without any additives; group 2: basal diet mixed with garlic (40 g/kg diet); group 3: basal diet mixed with ascorbic acid (1.5 g/kg diet); group 4: basal diet mixed with a combination of both garlic (20 g/kg diet) and ascorbic acid (0.75 g/kg diet). TEC: total erythrocytic count; PCV: packed cell volume; Hb%: hemoglobin percentage; MCV: mean corpuscular volume; MCH: mean corpuscular hemoglobin; MCHC: mean corpuscular hemoglobin concentration.

**Table 6:** Influence of garlic and/or ascorbic acid on biochemical parameters of juvenile barramundi (Asian sea bass, *Lates calcarifer* Bloch) fish.

Parameters	Groups			
	Group 1	Group 2	Group 3	Group 4
Glucose	13.1 ± 0.8	12.5 ± 0.6	10.0 ± 0.5	9.9 ± 0.6
Total protein (g/L)	4.1 ± 0.1	4.3 ± 0.1	4.2 ± 0.1	3.9 ± 0.1
Albumin (g/L)	1.5 ± 0.1	1.6 ± 0.1	1.7 ± 0.1	1.7 ± 0.1
Globulin (g/L)	2.6 ± 0.1	2.7 ± 0.1	2.5 ± 0.1	2.2 ± 0.3
A/G ratio	0.6 ± 0.1	0.6 ± 0.1	0.7 ± 0.1	0.8 ± 0.2
Total cholesterol (mg/dL)	210.3 ± 7.3 ^a^	190.2 ± 5.2 ^b^	206.1 ± 5.1 ^a^	204.2 ± 5.3 ^a^
ALT (U/L)	12.5 ± 0.5	11.9 ± 0.5	12.1 ± 0.5	12.5 ± 0.5
AST (U/L)	29.2 ± 1.0	31.1 ± 1.4	0.3 ± 1.5	31.4 ± 1.6
ALP (U/L)	18.0 ± 2.6	22.5 ± 2.0	21.4 ± 2.5	18.5 ± 2.4
Bilirubin (mg/dL)	0.1 ± 0.02	0.1 ± 0.02	0.1 ± 0.03	0.1 ± 0.05
BUN (mg/dL)	1.7 ± 0.4	1.1 ± 0.3	1.1 ± 0.4	1.5 ± 0.4
Uric acid (mg/dL)	1.6 ± 0.1	1.7 ± 0.1	1.7 ± 0.1	1.6 ± 0.1
Calcium (mg/dL)	11.9 ± 1.2	11.7 ± 1.0	12.6 ± 1.1	12.5 ± 1.0
Phosphorus (mg/dL)	10.8 ± 2.0	11.0 ± 1.5	10.6 ± 1.4	10.0 ± 1.5
Magnesium (mg/dL)	1.2 ± 0.2	0.9 ± 0.1	1.0 ± 0.1	1.0 ± 0.1
Chloride (mEq/L)	163.6 ± 2.2	163.7 ± 2.0	165.8 ± 2.3	160.1 ± 2.5

Values are mean ± standard error (SE) of three replicates of fish (*n* = 3). ^a,b^ values with different superscripts in the same row significantly differed at *p* < 0.05. Group 1: basal diet without any additives; group 2: basal diet mixed with garlic (40 g/kg diet); group 3: basal diet mixed with ascorbic acid (1.5 g/kg diet); group 4: basal diet mixed with a combination of both garlic (20 g/kg diet) and ascorbic acid (0.75 g/kg diet). A/G ratio: albumin/globulin ratio; AST: aspartate transaminase; ALT: alanine transaminase; ALP: alkaline phosphatase; BUN: blood urea nitrogen.
